# Evaluation of the Bioavailability and Translocation of Selected Heavy Metals by *Brassica juncea* and *Spinacea oleracea* L for a South African Power Utility Coal Fly Ash

**DOI:** 10.3390/ijerph15122841

**Published:** 2018-12-13

**Authors:** Aluwani Shiridor Mashau, Mugera Wilson Gitari, Segun Ajayi Akinyemi

**Affiliations:** 1 Environmental Remediation and Nanoscience Group, Department of Ecology and Resources Management, University of Venda, Private Bag X5050, Thohoyandou 0950, South Africa; shirldor.mashau03@gmail.com; 2 Environmental Remediation and Geopollution Group, Department of Geology, Faculty of Science, Ekiti State University, Ado Ekiti, Private Mail Bag 5363, Ado Ekiti 360001 Nigeria; segun.akinyemi@eksu.edu.ng

**Keywords:** coal fly ash, leachates, chemical species, pot-culture experiments, translocation, bioconcentration

## Abstract

This study evaluated the physicochemical and mineralogical properties, mobile chemical species bioavailability and translocation in *Brassica juncea* and *Spinacea oleracea* L. plants of a South African coal-fired power utility. Coal-fly-ash (CFA) disposal is associated with various environmental and health risks, including air, soil, surface, and groundwater pollution due to the leaching of toxic heavy metals; these ends up in food webs affecting human health, while repeated inhalation causes bronchitis, silicosis, hair loss, and lung cancer. The morphology and chemical and mineralogical composition of CFA were determined using Scanning Electron Microscopy (SEM), X-ray fluorescence (XRF), and X-ray diffraction, respectively. In pot-culture experiments, *S. oleracea* L. and *B. juncea* plants were grown in three sets of pots containing CFA (Set 1), soil (Set 2), and a mixture of CFA plus soil at a ratio of 1:1 (50% CFA: 50% soil, Set 3), while no plants were grown in Set 4 as a control for the leachate samples. SEM showed that the surface morphology of CFA has a lower degree of sphericity with the irregular agglomerations of many particles. XRF results revealed that CFA contains 43.65%, 22.68%, and 10.89% of SiO_2_, Al_2_O_3,_ and Fe_2_O_3,_ respectively, which indicates that CFA is an aluminosilicate material. X-ray diffraction (XRD) showed that CFA contains mullite as a major phase, followed by quartz mineral phases. Chemical species such as B, Ba, Mo, and Cr were occurring at higher concentrations in the leachates for most weeks in the pot-culture experiments, especially for CFA and soil + CFA growth media. However, there was a common trend for all growth media of chemical-species concentrations declining with time, which might have been caused by plant uptake or wash-off with water during irrigation; even for the growth media as well, where no plants were grown. Chemical species, such as Fe, Mn, B, Ba, and Zn, accumulated highly in most parts of the plant species. However, *B. juncea* showed higher potential to accumulate chemical species as compared to *S. oleracea* L. Bioconcentration and translocation factors (BF and TF) showed that *B. juncea* was the most effective in terms of bioconcentration and translocation of most of the chemical species. This indicates that *B. juncea* has potential in application for the phytoremediation of CFA dumps, and could contribute to the remediation of CFA dumps and the reduction of potential health and environmental impact associated with CFA.

## 1. Introduction

Coal-fly-ash dumps are a major source of chemical-species contamination of the environment as a whole. Maiti and Prasad [1] mentioned that coal-based thermal power plants generate coal fly ash (CFA) as the main industrial-waste product, at approximately 70%–75% [2], and it has been recognized as an environmental hazard across the globe. In South Africa, coal-fired power stations consume ±120 million tons of coal per annum, producing 30 million tons of CFA, to supply the bulk of South Africa’s electricity. A modern coal-fired power station, with a total output of 3600 MW, was said to consume ±50,000 tons of coal every day by Eskom [3]. Eskom [3] mentioned that, depending on coal quality, heat, and ash content, stations can produce ±17,000 tons of ash per day. On the other hand, Sasol was producing about 7 million tons of ash, as reported by Reference [4]. Many of South Africa’s thermal power plants use a wet-ashing process, while others use dry-ashing processes to dispose of ash. Usually, CFA is transported from the power plant, either damp or as a slurry, to a series of holding ponds where solids are allowed to settle out of suspension. CFA is usually then stockpiled and used as landfill. In dry-ashing systems, CFA is transported from the boilers by overland conveyors to ash-disposal facilities, and, since ash contains 12% moisture, dust production is minimal [5].

Even though that is the case, agricultural lands have been continuously contaminated by direct discharge of industrial effluents, runoff wastewater from ash dumps, overflow of ash dykes during rainy seasons, or through the atmospheric fallout of CFA [6]. If soil and water get contaminated with industrial effluents containing high levels of chemical species, the chemical species find their way into food crops and vegetables and, consequently, enter the food web. Most of these chemical species are toxic and persistent at high concentrations, while some are toxic even at trace concentrations. They are regarded as environmental pollutants and pose a threat to the environment and human health [1]. Thus, improper CFA dumping will continue to cause land degradation, water, air, and soil pollution if preventive measures are not implemented. Dust emission of CFA causes air pollution and remains airborne for a long period, causing health hazards for the local masses. Henceforth, repeated inhalation of CFA dust containing crystalline silica can cause bronchitis, silicosis (scarring of the lung), lung cancer, and severe inflammation of the small airways of the lung, causing asthmalike symptoms. Exposure to some of the most common toxic contaminants in CFA, like Ni, causes allergic dermatitis known as nickel itch; inhalation can cause cancer of the lungs, nose, and sinuses; cancers of the throat and stomach have also been attributed to its inhalation; it is hematotoxic, immunotoxic, neurotoxic, genotoxic, reproductive-toxic, pulmonary-toxic, nephrotoxic, and hepatotoxic; it also causes hair loss [7].

Phytoremediation is a way to go about this pollution of air, soil, and water sources caused by CFA, where chemical species also end up in the food chain, affecting human life; it is a cost-efficient and ecologically benign process. This is because conventional remediation methods, such as acid leaching, land-filling, and excavation processes, are very expensive and not ecofriendly [8]. Phytoremediation is aimed at providing an innovative, economical, environmentally friendly approach for removing toxic chemical species from hazardous-waste sites [6]. It involves techniques such as rhizofiltration, phytostabilization, phytovolatilization, and phytoextraction. Emphasis is on phytoextraction because it involves chemical-species translocation to shoots, which is an important biochemical process and advantageous in effective phytoremediation. In other techniques, the harvest of root biomass is generally not feasible in many instances [7,9,10]. Goswami and Das [11] studied phytoremediation of cadmium-contaminated soil using *B. juncea,* and bioaccumulation was observed to be effective in the decontamination of the soil. Other plant species, like *Ipomea carnea* [12], *Jatropha curcas* [13], and *Azolla caroliniana* [14], are some that have been evaluated for the phytoremediation of CFA dumps. *B. juncea* and *S. oleracea* L. were chosen for the current study because they have certain characteristics that make them suitable for phytoremediation applications. This includes having a high growth rate, producing more aboveground biomass, accumulating more of the target chemical species from the soil, translocating accumulated chemical species from roots to shoots, tolerating the toxic effects of the target chemical species, adaptating well to prevailing environmental and climatic conditions, and being easy to cultivate and harvest [7,15,16,17].

The aim of this study was to determine the potential of *S. oleracea* L. and *B. juncea* in the phytoremediation of chemical species from CFA dumps. Therefore, the specific objectives were: (i) to evaluate the physicochemical and morphological composition of CFA from a selected South African coal-fired power utility, and (ii) to assess the bioavailability and translocation factor of chemical species in *S. oleracea* L. and *B. juncea* over a specific period of time and their potential for field application in the phytoremediation of such CFA dumps. There is a gap in the field of phytoremediation of CFA dumps, and most studies have focused more on the phytostabilization than the phytoextraction of CFA dumps, hence showing the significance of the current study.

## 2. Materials and Methods

### 2.1. Materials

The dry CFA used was collected from a selected South African coal-fired power utility. Good and viable seeds of *Spinacia oleracea* L. and *Brassica juncea* were purchased from a local store, sown in seed trays, and, thereafter, transplanted to pots for the growth experiments.

### 2.2. Physicochemical Characterization of Coal Fly Ash

The chemical and mineralogical composition of the CFA and soil were determined using X-ray fluorescence (XRF, ThermosFisher ARL Perform’X Sequential XRF instrument, Switzerland) and X-ray diffraction (XRD, Bruker, Germany), respectively. XRD analysis was done using a PANalytical X’Pert Pro powder diffractometer in θ–θ configuration with an X’Celerator detector, variable divergence, and fixed receiving slits with Fe-filtered Co–Kα radiation (λ = 1.789 Å). Scanning Electron Microscopy (FEI Nova NanoSEM 230 with field emission gun, Eindhoven, Netherlands) was used to examine the morphology of CFA, and a Zeiss 1450 fully analytical scanning electron microscope was used.

### 2.3. Experimental Design

**Figure 1:**
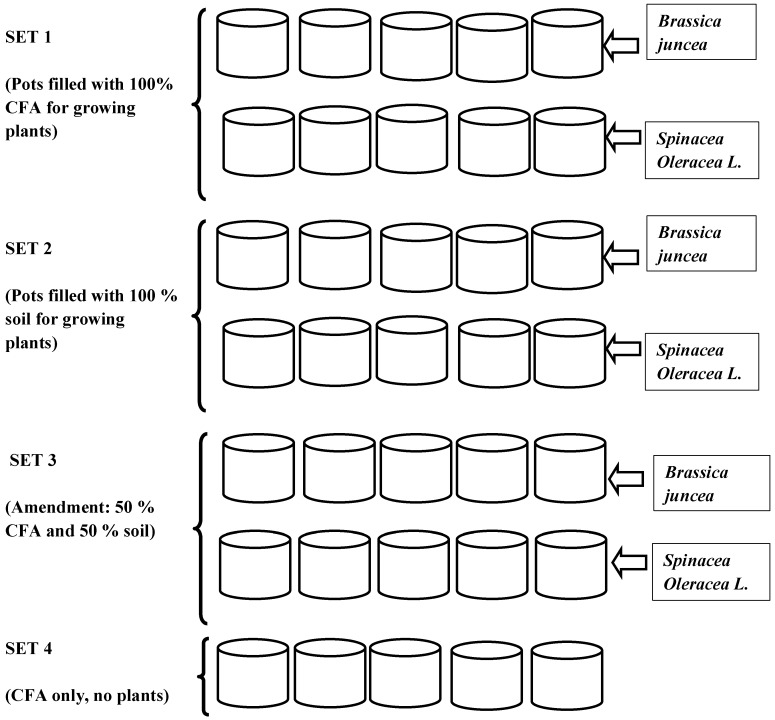
Illustration of pot setup (materials used in each pot and ratio used in case of combination is indicated; species grown in each pot is also shown). CFA: Coal-fly-ash.

Pot-culture experiments were conducted for four successive months (April to July 2015). Prior to the experiment, the pots were thoroughly washed with Milli-Q water and filled up with different compositions of soil and CFA. Experiments were conducted in four sets. The first set was filled up with CFA, second set with soil only, the third set with a mixture of CFA and soil, and the last set was for control with only CFA. *Spinacia oleracea* L. and *Brassica juncea* plants were grown in three sets of pots, containing CFA (Set 1), soil (Set 2) and a mixture of CFA and soil at ratio 1:1 (50% CFA: 50% soil, Set 3). No plant was grown in Set 4. Figure 1 shows the sets of used growth media. Seeds were sown in seed trays and irrigated daily until germination, then transplanted to pots. The pots were kept in a nursery to mimic the natural environment. The pots were monitored (measuring plant heights for growth performance) and leachates were collected daily; finally, the heights of the plants were recorded. Plants were irrigated daily and the collected leachate was analyzed using Inductively Coupled Plasma-Mass Spectrometry (Agilent 7900 ICP-MS, Agilent Technologies, Santa Clara, CA, US) for cationic chemical species. Harvesting of the plants was done in stages: the first harvest was of the seedlings (23 days), the second harvest was done after 69 days of growth, and the last harvest was done after 115 days of growth. This was chosen based on the stages of maturity of the two plant species; 23 days for the seedlings was when they were transplanted to bigger pots, 69 days as when the plants began to mature, and 115 days was when plants were fully matured.

### 2.4. Preparation of Plant Samples for Bioaccumulation Analysis, Biomass Estimation, and Chlorophyll Content

Samples of the whole plant were collected, then cut to separate parts, leaves, stems, and roots, in order to determine chemical-species concentrations in each part of the plant and for biomass estimation. The biomass of the plants was estimated by weighing the mass (g) of plant sample after air-drying. To estimate chemical-species bioaccumulation, dried samples were ground into a fine powder, and 0.5 g was weighed for digestion through aqua regia (HCl:HNO_3_ = 3:1 (*v*/*v*)) to near dryness or until a white-colored solution was formed. Acid digestion was carried out on a hotplate. After complete digestion of the samples, 100 mL of MilliQ water (18.2 MΩ/cm) was added and left to cool down. Samples were then filtered through a 0.45 µm pore membrane. Samples were then analyzed using ICP-MS (Agilent 7900 ICP-MS, Agilent Technologies, Santa Clara, CA, US). Blanks and internal standards were set for quality assurance. Growth performance was obtained by measuring the heights of the plant species in the pot-culture experiments.

For chlorophyll analysis, the leaves of *B. juncea* and *S. oleracea* L. were accurately weighed, and 0.5 g of each fresh plant leaf sample was taken. This was sufficiently homogenized in a blender with 10 mL of the extracting solvent (90% ethanol). The homogenized sample mixture was centrifuged at 10,000 rpm for 15 min at 40 °C. The supernatant was separated, and 0.5 mL of it was mixed with 4.5 mL of ethanol. The solution was transferred to a corresponding cell, put in a cell compartment, and analyzed for Chlorophyll-a, Chlorophyll-b, and carotenoid content using a spectrophotometer (SQ Pharo 100 Spectroquant, Merk, Frankfurt, Germany). The chlorophyll contents were then determined using the following equations [18]:

(1) $$\mathrm{Ch}-a=13.36A_{664}-5.19A_{649}$$

(2) $$\mathrm{Ch}-b=27.43A_{649}-8.12A_{664}$$

(3) $$\mathrm{Cx}+c=\left(1000A_{470}-2.13\mathrm{Ch-a}-97.63\mathrm{Ch-b}\right)/209$$

where A = Absorbance, Ch-a = Chlorophyll a, Ch-b = Chlorophyll b, and C x+c = Carotenoids.

### 2.5. Bioconcentration Factor (BCF) and Translocation Factor (TF)

BCF was calculated for each plant part (root, stem, leaf) using the following equations for the CFA and soil:

(4) BCFa= Metal in leaves / Metal in FA or soil.

(5) BCFb = Metal in stem / Metal in FA or soil.

(6) BCFc = Metal in roots / Metal in FA or soil.

where BCFa is the bioconcentration factor of the leaves, BCFb is the bioconcentration factor of the stem, and BCFc is the bioconcentration factor of the roots.

Then, TF, which is an asset to assess a plant’s potential for phytoremediation purposes, was also calculated. TF is based on the ratio of metal concentration in a plant stem as compared to that of the plant root and leaves [19].

Thus:

(7) TF = BCFa / BCFb i.e., leaf/stem.

(8) TF = BCFb / BCFc i.e., stem/root

### 2.6. Statistical Analysis and How Chemical-Species Concentrations Were Calculated in Plant Parts after Analysis

Data were subjected to one-way analysis of variance (ANOVA) using a Graphpad software package (http://www.graphpad.com). The relationship between chemical elements in soil, FA, and FA + soil growth media, and also between the different plant parts (root, stem and leaf) of *B. juncea* and *S. oleracea* L., were calculated using the *t*-, ANOVA–Bartlett, and Mann–Whitney tests, and Kruskal–Wallis Statistics (KW) depending on each dataset. This was done to determine how well the datasets were related to each other with regards to the concentrations of chemical elements in each plant part and also in different growth media. Differences were considered significant at *p* ≤ 0.05.

The dry-weight concentrations of the chemical elements in the plant parts were calculated using the following formula for both *B. juncea* and *S. oleracea* L.:

$$PPM\;=\;\frac{C\times V}{W}$$

where

*C* = concentration value from ICP-MS;
*V* = volume of the solution used in the analysis; and
*W* = weight of the plant part used.

## 3. Results and Discussion

### 3.1. Physicochemical Characterization of a Selected South African Coal-Fired Power Utility

**Figure 2:**
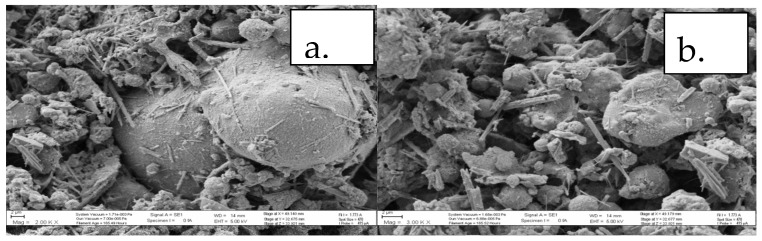
Scanning electron microscopy (SEM) micrographs of selected South African coal-fired power-utility coal fly ash (CFA) at (**a**) ×20,000 and (**b**) ×30,000 magnifications.

Figure 2 depicts the morphology of CFA as determined by SEM at different magnification levels. It was observed that morphology of CFA had a lower degree of sphericity with irregular agglomerations of many particles, while there were dominant spherical particles and smaller sharp needlelike particles.

**Figure 3:**
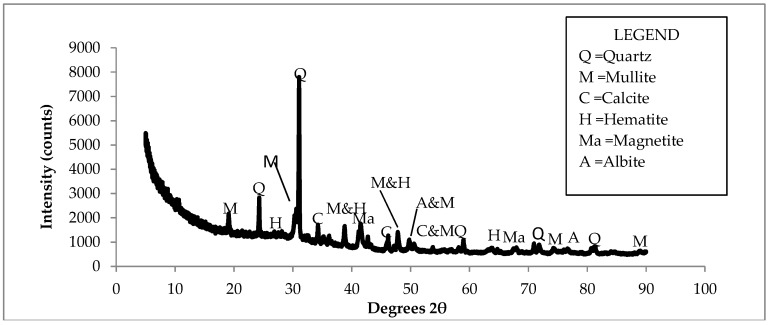
X-ray diffraction (XRD) spectrum of the CFA.

Figure 3 presents the XRD spectrum of the CFA. The spectra showed the presence of mullite, quartz, calcite, hematite, magnetite, and albite as mineral phases in the CFA. Quantitative results from XRD showed that mullite was the dominant mineral (48.14%), followed by quartz (28.51%). Other mineral phases were at trace levels.

Table 1 shows the chemical composition of CFA and soil as determined by XRF. The analysis revealed that CFA consisted of Fe_2_O_3_ (10.89%), SiO_2_ (43.65%), and Al_2_O_3_ (22.68%)_._ The total percentage of these three oxides was >70%, indicating that this South African coal fly ash can be categorized as class F (SiO_2_-rich), which is derived either from anthracitic or bituminous coals [20]. The high concentration of Fe_2_O_3_, SiO_2,_ and Al_2_O_3_ confirms that this CFA is an aluminosilicate material [21]. The soil had SiO_2_ (82.9%) as the main component, while Fe_2_O_3_ and Al_2_O_3_ were available in small amounts. Elements such as Ni, Cu, Zn, Zr, W, Sr, Ni, As, Rb, and Mo were observed at trace levels in both CFA and soil.

### 3.2. Assessment of Biomass, Growth Performance, and Chlorophyll Content of Plant Species in Pot-Culture Experiments

**Figure 4:**
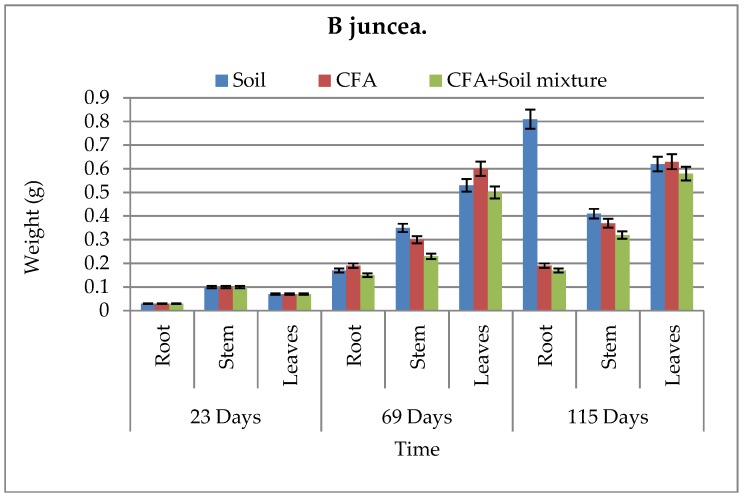
Above- and below-ground biomass for *B. juncea* in all growth media. CFA: Coal-fly-ash.

**Figure 5:**
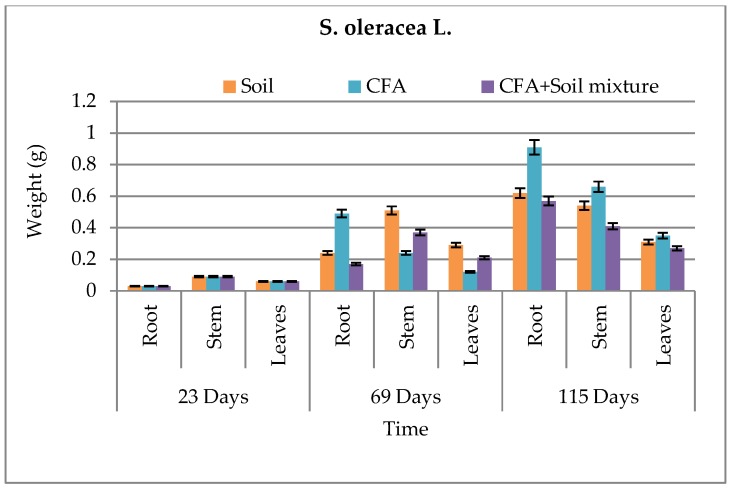
Above- and below-ground biomass for *S. oleracea* L. in all growth media. CFA: Coal-fly-ash.

**Figure 6:**
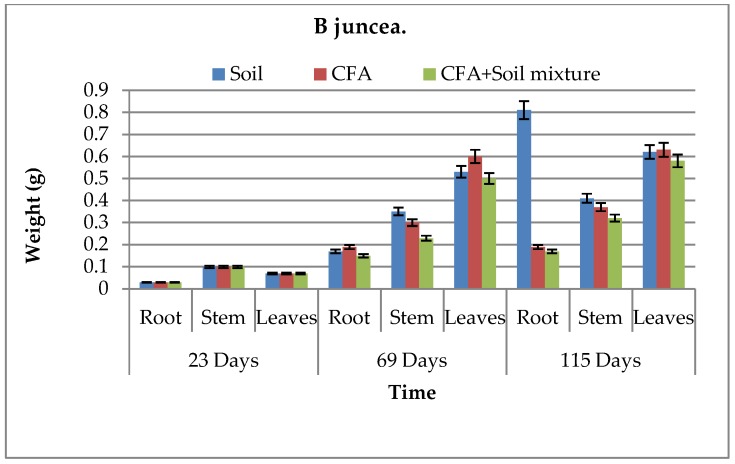
Growth performance of *B. juncea* in all growth media. CFA: Coal-fly-ash.

**Figure 7:**
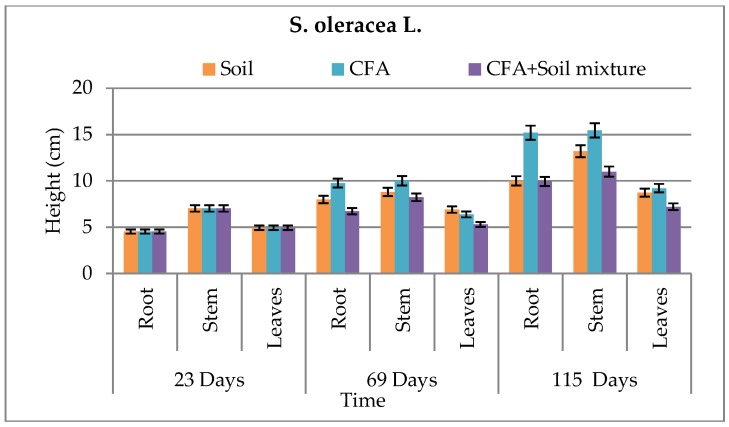
Growth performance of *S. oleracea* L. in all growth media. CFA: Coal-fly-ash.

The biomass and growth performance of *B. juncea* and *S. oleracea* L. over time in different growth media are presented in Figure 4, Figure 5, Figure 6 and Figure 7, respectively. Results showed that, in both growth media, the root, stem, and leaf biomass of *B. juncea* and *S. oleracea* L. plant species increased with increasing number of days. Based on the root and stem mass, *S oleracea* L. showed better tolerance in CFA as compared to *B. juncea* in CFA growth media. It is, therefore, anticipated that *S. oleracea* L. would yield better performance in phytoremediation because plants with extensive roots are capable of extracting more chemical species due to better exploration of growth media [13]. Furthermore, *S. oleracea* L. showed better growth in CFA media, while *B. juncea* grew better in the soil as a growth medium.

It was observed that chlorophyll a and b increased during the first 69 days and declined in 115 days for some of the growth media (Table 2). This was observed in both plant species. Goswami and Das reported similar findings, where there was significant reduction in the total chlorophyll content of the young leaves of *B. juncea* and *B. napus* on exposure to 10 mM Cd for 15 days [11]. Compared to *B. juncea*, *S. oleracea* L. showed better chlorophyll b content. Even though that is the case, *B juncea* showed to have more survival characteristics by having some carotenoid content while *S. oleracea* L.’s was not detected (Table 2). This carotenoid content in *B. juncea* plants can help in the protection of the plant species and improve its tolerance to harsh environments, including the high amounts of chemical species found in CFA; hence, it can promote its effectiveness in phytoremediation better than *S. oleracea* L. In an excess of light, carotenoids play an important role in photoprotection, and also have ecological significance [22]. Carotenoid content also protect plants against overexcitation in strong light, and dissipates excess absorbed energy; carotenoids scavenge reactive oxygen species formed during photo-oxidative stress and moderate the effect of extreme temperatures [23]. However, statistically, there was no significant difference between the two plant species in terms of growth performance and biomass, even between the plant parts denoting similar growth performance for the two plant species under study. This was because the P values for both species relative to the three different sets of growth media were greater than 0.05, hence showing no significant difference. These statistical results confirm that the little difference we saw in Figure 4, Figure 5, Figure 6 and Figure 7 was just not significant enough to have an effect on the biomass and growth performance of the plants under study. This might have been influenced by similar characteristics that the two plant species host, and the similar characteristics possessed by the soil and CFA used as the growth media for the pot-culture experiments.

### 3.3. Temporal Evolution of the Physicochemical Characteristics of the Leachates as Plants Irrigated in Pot-Culture Experiment

The temporal variation of the chemical elements in the leachates from different growth media is based on results for leachates collected from the first to the eleventh week of the pot-culture experiments for the different growth media. The full analysis results are presented in the supplementary information in Tables S1–S7. The selected chemical species presented include B, Cr, Mn, Ni, Cu, Zn, Mo, Ba, and Fe. They were chosen based on their higher concentrations in the leachates. Several authors have also indicated that these chemical species were present in CFA leachates in higher concentrations [8,12,24].

**Figure 8:**
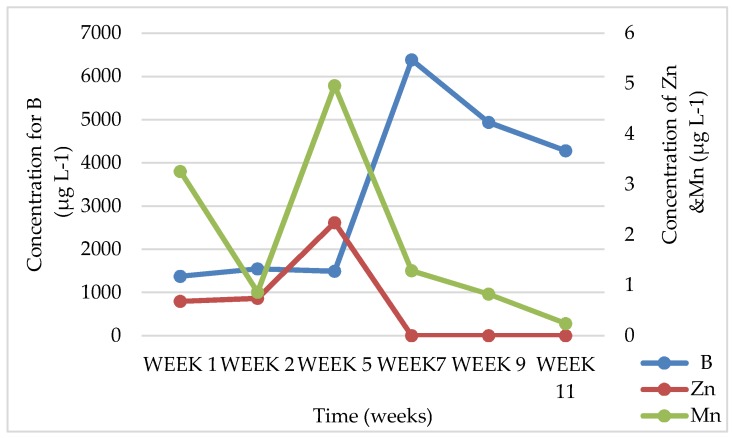
Chemical-element concentrations in leachates obtained from CFA (Coal-fly-ash) pots growing *S. oleracea* L. for essential elements B, Zn, and Mn.

**Figure 9:**
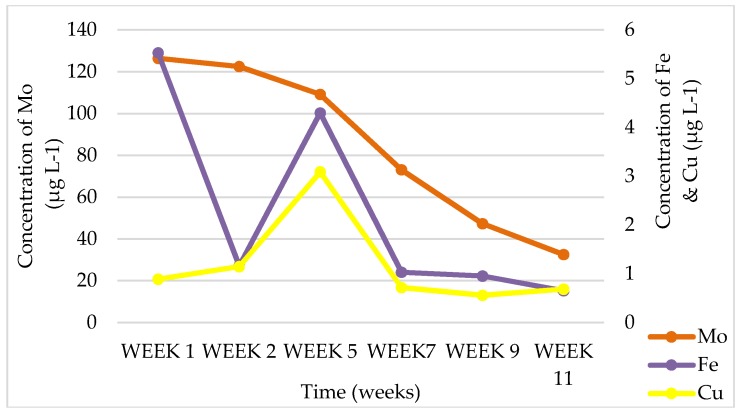
Chemical-element concentrations in leachates obtained from CFA (Coal-fly-ash) pots growing *S. oleracea* L. for essential elements Fe, Mo, and Cu.

**Figure 10:**
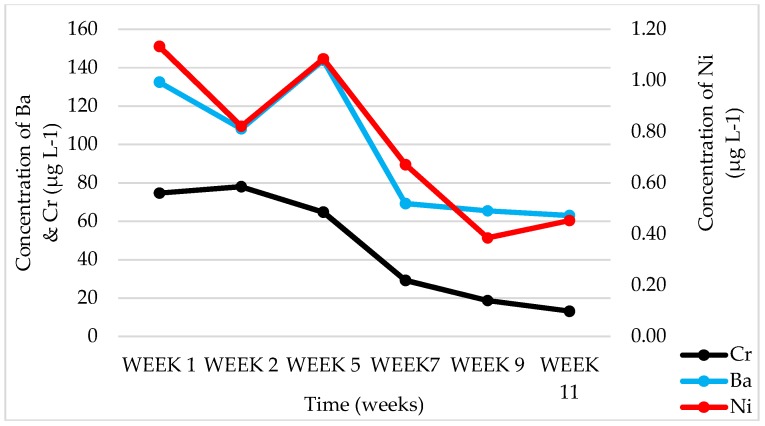
Chemical-element concentrations in leachates obtained from CFA (Coal-fly-ash) pots growing *S. oleracea* L. for nonessential elements Cr, Ni, and Ba.

In batch-leaching tests, soil leachates had a neutral pH of 7.22 with very high amounts of Si CFA leachates, meanwhile, had an alkaline pH of 10.62, which is known to be due to low sulfur content [14,25,26,27], and this can be seen in Table 1. In both soil and CFA, chemical species that were expected to be readily available to plants included Ca, Si, K, Ba, Mo, Na, Al, Mg, and Sr, where Si, Ba, Na, Al, and Sr are nonessential elements as they were found in leachate solutions, which, if uptaken by plant roots, can have a negative impact on the plants. Physicochemical analysis of the soil, CFA, and CFA + soil leachates where plants were grown and where there were no plants showed that the alkalinity of the CFA changed over time (starting from an average of 9.4 to 7.5 pH level in the 11th week) and there was also a decrease in the EC (starting from an average of 411 to 224 µS cm^−1^ in the 11th week) due to the dissolution of soluble major oxides, which was promoted by continuous irrigation with water. Chemical species like B, Cr, Mo, and Ba (ranging from 4277.50 to 13.04 µg L^−1^) occurred at higher concentrations in leachates for most weeks for CFA and soil + CFA as a growth medium, while Fe (starting from 10,003.41 to 550.25 µg L^−1^ in the 11th week) was high for soil as a growth medium. It was observed that, in the 11th week of leachate collection, all these chemical species plummeted to very low concentrations. An example of the chemical elements that were found to be bioavailable in CFA leachate samples for *S. oleracea* L. can be seen in Figure 8, Figure 9 and Figure 10. This suggests that these chemical elements could be reduced over time as plants are irrigated, which is either due to uptake by plants, or being washed off with water and/or CFA weathering processes [14]. A similar trend was observed for CFA, soil, and CFA + soil growth media. There was a significant difference in the concentrations of different chemical species in leachates from different growth media for each plant species (*B. juncea* and *S. oleracea* L.).

**Figure 11:**
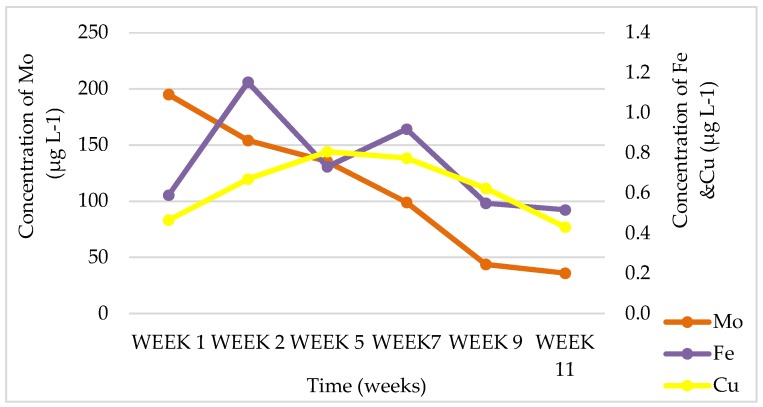
Chemical-element concentrations obtained from CFA (Coal-fly-ash) pots where no plants were grown for essential elements Fe, Mo, and Cu.

Figure 11 shows three out of the nine chemical elements that were found to be bioavailable in CFA where no plants were growing.

Only this figure was presented because a similar trend can be observed for the other six chemical elements. Even here (just like in the pots were plant species were grown), the trend shows that chemical-element concentrations start high or increase during the earlier weeks, but at the final week (Week 11), we found them decreased in concentration. This marked similarity might be associated with the pH levels that also show some similarity in leachates for the different observed weeks. Pandey also mentioned that the chemical composition of CFA leachates is affected by the weathering of CFA over time, resulting in lower concentrations in the final week (Week 11) of the collected leachates for the current study [14].

### 3.4. Potential of Plant Species for Phytoremediation

The phytoremediation potential of a plant is assessed by computing the BCF and TF of the chemical species throughout the plant. A BCF value of more than 1 indicates that the plant is a potential accumulator of chemical species, while a TF value greater than 1 indicates that the plant is a potential translocator of chemical species. These also show the role and importance of plant species used for the current study on the uptake and translocation of chemical elements to shoots, allowing detoxification in the process without affecting the physiology of the plants.

Table 3 shows the BCF for chemical species accumulating in *B. juncea* and *S. oleracea* L. on Day 115 for all growth media. Higher BCF values were observed for Fe, Mn, Zn, Cu, and Ni in the plant parts of both plants. Conversely, lower values were observed for Mo, B, Ba, and Cr, having BCF values less than 1 for most growth media over time. From the results, it was concluded that *B juncea* is more suitable for the accumulation of many chemical species than *S. oleracea* L. in CFA and soil as a growth medium (Table 3). Chemical-species accumulation trends depend on factors such as the age and type of the plant, chemical-species concentrations in CFA, and also the season of sampling [14]; this is the reason why accumulation was different over time for the current study (See details on 69 days in Table S8 in the supplementary materials). For the CFA + soil-growth media, *B juncea* accumulated many different chemical species than *S. oleracea* L. in 115 days. This is the growth medium where *S. oleracea* L. did better than other growth media in terms of the accumulation of different chemical species. Similar results were obtained by Pandey, but using a different plant species for the phytoremediation of CFA dumps. The results indicated the efficiency of *Azolla caroliniana* for the phytoremediation of the CFA pond because of its higher bioconcentration factor. The metal concentration ranged from 175 to 538 and 86 to 753 mg kg^−1^ in roots and fronds, respectively [14].

Table 4 shows the translocation factor of various chemical species over time (115 days) in different plant species grown in different growth media with respect to the translocation of chemical species from the roots to the shoots of the plant species under study. The *B. juncea* species was observed to be an effective translocator of various chemical species (including Zn) for different growth media. In most cases, TF values > 1 were observed for *B. juncea,* while *S. oleracea* L. failed to translocate most chemical species (TF < 1). Translocation was significant from the stems to the leaves of *B. juncea* in soil-growth media, followed by CFA, and then lastly by CFA + soil-growth media. Hence, it could be a potential CFA-dump phytoremediator. There was also significant chlorophyll and carotenoid content in *B. juncea,* leading to better tolerance in CFA media, while *S. oleracea* L. did not possess any carotenoid content that might have weakened its chemical-species accumulation potential. Goswami and Das studied the cadmium phytoremediation potential of *B. juncea*, and calculated the enrichment coefficient and root to shoot translocation factor that pointed toward the suitability of *B. juncea* for removing Cd from soil [11]. Hence, *B. juncea* has the potential of accumulating many different pollutants, just like in the current study, where nine chemical elements were removed and translocated to the shoots. In the other study, it was suggested that *Jatropha curcas* has the potential to establish itself on CFA when provided with basic plant nutrients, and can manifold accumulating heavy metals from FA without attenuating plant growth [13].

The differences in the metal concentrations of the plant parts (root, stem, leaf) suggest different cellular mechanisms of bioaccumulation that may manage and control their translocation and partitioning in plant systems [12,28]. Soluble chemical elements can enter roots either via cell-wall free space (apoplastic pathway) or by transport, across the plasma membrane of root cells and movement through the cytoplasm (symplastic pathway). They may also travel across the cell and ultimately enter the translocation stream [11].

## 4. Conclusions

The findings of the current study show that CFA is an aluminosilicate material with an alkaline pH. CFA morphology showed that it has a lower degree of sphericity with irregular agglomerations of many particles, while there were dominant spherical particles and smaller, sharp, needlelike particles. There was similarity in terms of the elemental composition of CFA and soil used in this study since SiO_2_ was available in both media as a major oxide, which gives an expectation of similar growth of the plants in both media. Chemical species, such as B, Ba, Mo, and Cr, occurred at higher concentrations in the leachates for most weeks in the pot-culture experiments, especially for CFA and soil + CFA growth media. Fe also dominated, but only in soil as a growth medium. In terms of biomass estimation, growth performance, and the chlorophyll content of both plants, *S. oleracea* L. showed better growth in CFA media, while *B. juncea* grew better in the soil as a growth medium. It was observed that chlorophyll a and b increased during the first 69 days, and declined in 115 days for some growth media. Furthermore, there was some carotenoid content found in *B. juncea,* while none was detected for *S. oleracea* L. Because carotenoid content is important in the protection of plant, this could be attributed to the better adaptability of *B. juncea* for growth in CFA, and being a better bioaccumulator and translocator of chemical species. Hence, it could be more effective plant for phytoremediation than *S oleracea* L. The superiority of *B. juncea* was confirmed with a BCF and TF >1 for most chemical species as compared to *S. oleracea* L. This study observed that *B. juncea* is a plant species that can potentially be applied on a field scale for the phytoremediation of CFA dumps. This could significantly lead to the reduction of the environmental and health impact of coal fly ash dumps, especially for communities leaving near such coal-fly-ash storage facilities. However, there is still a need for the assessment of other plant species.

## Figures and Tables

**Table 1:** Comparison of the chemical composition of CFA and soil samples.

CFA Sample				Soil Sample			
Major Elements (as Oxides)	(*w*/*w*) %	Trace Elements	mg kg^−1^	Major Elements (as oxides)	(*w*/*w*) %	Trace Elements	mg kg^−1^
SiO_2_	43.65	As	22	SiO_2_	82.90	As	24
TiO_2_	1.23	Cu	57	TiO_2_	0.54	Cu	17
Al_2_O_3_	22.68	Ga	35	Al_2_O_3_	7.33	Ga	9
Fe_2_O_3_	10.89	Mo	9	Fe_2_O_3_	3.31	Mo	8
MnO	0.06	Nb	31	MnO	0.03	Nb	9
MgO	1.88	Ni	63	MgO	0.25	Ni	20
CaO	7.04	Pb	29	CaO	0.36	Pb	6
Na_2_O	0.19	Rb	39	Na_2_O	0.71	Rb	51
K_2_O	0.81	Sr	2001	K_2_O	1.09	Sr	46
P_2_O_5_	0.54	Th	41	P_2_O_5_	0.05	Th	13
Cr_2_O_3_	0.18	U	28	Cr_2_O_3_	0.01	U	5
NiO	0.01	W	48	NiO	0.00	W	235
V_2_O_5_	0.02	Y	65	V_2_O_5_	0.01	Y	10
ZrO_2_	0.07	Zn	40	ZrO_2_	0.07	Zn	21
CuO	<0.01	Zr	507	CuO	0.00	Zr	405
SO_3_	0.36			LOI	3.02		
LOI	9.50			TOTAL	99.69		
TOTAL	99.11						

LOI: Loss On Ignition.

**Table 2:** Chlorophyll a and b content of *S. oleracea* L. and *B. juncea* for different growth media with time.

	Chlorophyll a for *B. juncea*			Chlorophyll a for *S. oleracea* L.		
	23 days	69 days	115 days	23 days	69 days	115 days
Soil	3.94	3.3	2.3	2.34	2.77	2.55
CFA	3.94	2.63	2.25	2.34	2.65	1.44
CFA + soil	3.94	4.11	3.94	2.34	2.64	2.75
	**Chlorophyll b for *B. juncea***			**Chlorophyll b for *S. oleracea* L.**		
Soil	0.87	1	0.8	4.95	6.08	5.51
CFA	0.87	1.57	1.79	4.95	5.62	3.12
CFA + Soil	0.87	0.93	0.87	4.95	5.86	5.95
	**Carotenoid content for *B. juncea***			**Carotenoid content for *S. oleracea* L.**		
Soil	0.92	0.59	0.69	ND	ND	ND
CFA	0.92	0.53	0.45	ND	ND	ND
CFA + Soil	0.92	0.76	0.92	ND	ND	ND

Note: ND = not detected; CFA: Coal-fly-ash.

**Table 3:** Bioconcentration Factor (BCF) for chemical species accumulating in both plant species on Day 115 for all growth media.

***B. juncea***									
**Growth Media**	**Soil**			**CFA**			**CFA + Soil**		
	**Leaf**	**Root**	**Stem**	**Leaf**	**Root**	**Stem**	**Leaf**	**Root**	**Stem**
B	2.67	1.01	2.40	0.12	0.08	0.06	0.07	0.02	0.03
Cr	5.66	4.37	2.06	1.00	1.08	0.55	2.13	0.58	0.65
Mn	24.00	15.79	7.72	309.84	200.39	262.18	315.57	118.69	106.95
Ni	1.20	1.32	0.59	24.57	24.32	11.75	10.66	3.47	3.28
Cu	6.53	5.96	2.46	27.58	19.96	25.26	9.31	4.44	3.78
Zn	16.43	28.56	11.90	185.73	181.15	196.67	85.29	44.32	35.85
Mo	7.29	1.12	3.24	0.28	0.15	0.15	0.03	0.02	0.03
Ba	2.72	2.36	1.86	1.22	1.10	1.38	1.32	0.50	0.52
Fe	68.31	50.51	17.89	979.08	692.36	1098.15	4046.11	1128.22	1047.44
***S. oleracea*** **L.**									
**Growth Media**	**Soil**			**CFA**			**CFA + Soil**		
	**Leaf**	**Root**	**Stem**	**Leaf**	**Root**	**Stem**	**Leaf**	**Root**	**Stem**
B	0.11	0.11	0.11	0.01	0.01	0.01	0.01	0.01	0.01
Cr	1.13	1.13	1.13	0.38	0.38	0.38	1.24	1.24	1.24
Mn	6.92	6.89	6.90	97.36	97.28	97.32	8.01	8.00	8.00
Ni	0.51	0.51	0.51	11.24	11.23	11.23	6.10	6.10	6.10
Cu	1.94	1.93	1.93	17.62	17.61	17.61	9.36	9.36	9.36
Zn	5.67	5.65	5.66	42.96	42.93	42.95	91.92	91.91	91.92
Mo	0.24	0.24	0.24	0.01	0.01	0.01	0.01	0.01	0.01
Ba	0.34	0.34	0.34	0.48	0.48	0.48	0.35	0.35	0.35
Fe	0.22	0.22	0.22	957.95	957.33	957.31	257.23	257.19	257.20

**Table 4:** Translocation Factor (TF) of various chemical species for 115 days in different species of plants grown in different growth media. TF = BCFb/BCFc, i.e., stem/root, and TF = BCFa/BCFb, i.e., leaf/stem.

	*B. juncea*						*S. oleracea* L.					
	Leaf/Stem			Stem/Root			Leaf/Stem				Stem/Root	
	Soil	CFA	CFA + Soil	Soil	CFA	CFA + Soil	Soil	CFA	CFA + Soil	Soil	CFA	CFA + Soil
**B**	3.57	5.15	1.79	2.37	0.74	1.33	1.06	0.68	2.75	1.21	1.85	0.77
**Cr**	4.84	3.32	1.03	0.47	0.51	1.13	2.55	0.25	0.45	0.77	1.35	0.66
**Mn**	3.86	4.44	1.08	0.49	1.32	0.91	0.63	18.75	3.70	0.45	0.05	0.59
**Ni**	4.04	2.90	1.01	0.45	0.48	0.94	0.98	1.58	0.57	0.46	0.16	0.40
**Cu**	4.35	3.34	1.20	0.41	1.27	0.85	1.17	1.75	0.62	0.61	0.24	0.42
**Zn**	4.22	3.26	1.24	0.42	1.09	0.81	0.80	0.44	1.42	0.55	1.25	0.65
**Mo**	8.95	5.46	3.36	2.88	0.97	1.72	1.72	1.30	3.59	0.76	1.12	0.66
**Ba**	2.64	3.10	1.22	0.79	1.26	1.05	0.60	1.50	1.16	0.65	0.38	0.56
**Fe**	5.38	3.68	0.91	0.35	1.59	0.93	2.39	2.80	0.59	0.60	0.17	0.61

## Supplementary Materials

The following are available online at http://www.mdpi.com/1660-4601/15/12/2841/s1, Table S1: Chemical-element concentrations in leachates obtained from soil pots growing *S. oleracea L.*, Table S2 Chemical-element concentrations in leachates obtained from CFA pots growing *S. oleracea L.*, Table S3: Chemical-element concentrations in leachates obtained from CFA + soil pots growing *S. oleracea L.*, Table S4: Chemical-element concentrations in leachates obtained from soil pots growing *B. juncea*., Table S5: Chemical-element concentrations in leachates obtained from CFA pots growing *B. juncea*., Table S6: Chemical-element concentrations in leachates obtained from CFA + soil pots growing *B. juncea.*, Table S7: Chemical-element concentrations in leachates obtained from CFA pots where no plants were grown, Table S8: BCF for chemical elements accumulating in *B. juncea* and *S. oleracea L.* from 69 to 115 days for all growth media.
