# Supplementary material for: Evaluation of the Bioavailability and Translocation of Selected Heavy Metals by Brassica juncea and Spinacea oleracea L for a South African Power Utility Coal Fly Ash
Source: Int J Environ Res Public Health. 2018 Dec 13;15(12):2841. doi: 10.3390/ijerph15122841 (PMC6313626; doi:10.3390/ijerph15122841)
Supplement: Supplementary file 1 [file ijerph-15-02841-s001.pdf]

Supplementary Materials

Table S1. Chemical elements concentrations in leachates obtained from soil pots growing *S. oleracea L.*

S. oleracea L.						
**Values in mg L^{-1}**						
**Soil media**	**WEEK 1**	**WEEK 2**	**WEEK 5**	**WEEK 7**	**WEEK 9**	**WEEK 11**
**Al**	14	7.38	8.22	6.22	3.14	1.28
**Ca**	25.78	23.30	4.74	109.90	3.26	1.05
**K**	29.44	27.45	9.30	48.88	7.52	0.69
**Mg**	15.22	8.85	2.39	60.79	2.45	0.33
**Na**	21.26	17.78	2.16	85.59	2.47	0.29
**P**	0.01	0.02	0.05	< 0.1	0.08	0.01
**Si**	14.65	16.13	11.25	167.90	32.96	0.95
**Sr**	0.14	0.37	0.03	0.75	0.03	0.01
**Values in µg L^{-1}**						
**Li**	<0.680	73.41	1.06	10.75	4.66	1.37
**Be**	0.01	0.11	0.07	1.34	0.42	<0.039
**B**	91.45	180.82	27.81	587.25	49.49	86.59
**Ti**	75.33	99.20	127.72	840.48	422.86	12.64
**V**	5.72	13.43	9.14	70.40	27.65	1.38
**Cr**	3.89	14.08	6.53	47.96	19.78	0.62
**Mn**	13.46	10.23	14.72	86.73	32.47	4.07
**Fe**	1997.88	3110.97	3564.41	39260.00	11790.00	297.21
**Co**	1.18	1.58	1.13	7.24	2.57	0.08
**Ni**	11.81	8.84	7.47	52.09	18.47	0.92
**Cu**	9.76	7.15	6.10	30.19	10.43	1.44
**Zn**	4.79	5.02	6.24	27.98	10.79	0.74
**As**	1.01	1.39	0.72	2.92	1.10	0.15
**Se**	1.48	1.84	0.66	2.06	<0.456	<0.456
**Mo**	0.22	14.16	0.24	0.52	0.21	0.21
**Cd**	0.03	0.03	0.00	0.03	0.02	0.01
**Sb**	0.12	0.34	0.09	0.22	0.11	0.04
**Ba**	136.54	92.90	54.80	402.76	133.97	5.50
**Hg**	0.01	0.01	<0.004	<0.003	0.00	0.00
**Pb**	0.40	0.49	0.55	6.56	1.70	0.05

Table S2. Chemical elements concentrations in leachates obtained from CFA pots growing *S. oleracea L.*

S. oleracea L.						
**Values in mg L^{-1}**						
**CFA media**	**WEEK 1**	**WEEK 2**	**WEEK 5**	**WEEK 7**	**WEEK 9**	**WEEK 11**
**Al**	0.28	2.45	0.91	0.42	0.30	0.27
**Ca**	116.70	124.40	154.90	147.70	96.80	71.71
**K**	66.39	64.01	31.97	8.86	7.55	6.24
**Mg**	4.40	4.96	8.66	46.05	32.10	24.12
**Na**	60.37	50.95	16.64	9.53	7.95	6.42
**P**	0.03	< 0.005	< 0.005	0.03	0.03	0.03
**Si**	1.65	0.76	1.89	1.92	1.87	1.89
**Sr**	3.33	3.53	4.39	3.06	1.95	1.54
**Values in μg L^{-1}**						
**Li**	711.93	595.96	302.86	346.20	260.15	226.12
**Be**	0.01	0.00	0.01	<0.039	<0.039	<0.039
**B**	1377.09	1550.94	1492.71	6382.09	4936.09	4277.50
**Ti**	0.22	0.08	0.26	0.08	<0.070	<0.070
**V**	68.47	81.70	64.09	25.84	25.43	24.68
**Cr**	74.64	78.00	64.76	29.15	18.70	13.16
**Mn**	3.26	0.87	4.96	1.29	0.83	0.24
**Fe**	5.53	1.17	18	1.04	0.96	0.65
**Co**	0.51	0.50	0.37	0.31	0.25	0.21
**Ni**	1.13	0.82	1.08	0.67	0.39	0.45
**Cu**	0.89	1.15	3.09	0.72	0.56	0.69
**Zn**	0.69	0.74	2.24	<0.181	<0.181	<0.181
**As**	9.63	8.17	4.34	5.90	5.95	5.47
**Se**	5.71	9.84	8.48	13.11	9.70	8.79
**Mo**	126.45	122.56	109.19	73.12	47.39	32.61
**Cd**	0.02	0.03	0.03	0.04	0.03	0.04
**Sb**	3.78	4.53	17	9.85	8.94	8.55
**Ba**	132.43	108.06	143.88	69.18	65.46	63.00
**Hg**	0.01	0.01	0.02	0.02	0.01	0.01
**Pb**	0.04	0.02	0.06	0.01	0.01	0.00

CFA: Coal-fly-ash.

Table S3. Chemical elements concentrations in leachates obtained from CFA + soil pots growing *S. oleracea L.*

S. oleracea L.						
**Values in mg L^{-1}**						
**CFA + soil media**	**WEEK 1**	**WEEK 2**	**WEEK 5**	**WEEK 7**	**WEEK 9**	**WEEK 11**
**Al**	0.03	0.03	0.03	0.03	0.02	0.02
**Ca**	235.30	226.10	135.20	68.88	50.29	6.27
**K**	42.39	37.46	25.42	3.46	7.04	0.52
**Mg**	25.77	21.71	18.55	23.72	15.71	1.40
**Na**	32.15	31.00	8.37	7.99	6.06	0.41
**P**	0.05	0.04	0.08	0.13	0.37	0.06
**Si**	14	5.05	3.05	4.69	3.60	0.60
**Sr**	3.66	3.42	2.23	1.70	1.11	0.13
**Values in µg L^{-1}**						
**Li**	25.32	20.34	52.78	35.08	19.00	3.20
**Be**	0.00	0.01	0.00	<0.039	<0.039	<0.039
**B**	2155.13	1762.79	1591.22	3110.40	1809.38	177.83
**Ti**	0.18	0.17	0.48	0.41	0.34	1.26
**V**	15.36	12.41	13.67	31.27	22.91	4.85
**Cr**	25.62	34.31	11.95	8.75	3.60	0.41
**Mn**	0.40	1.18	136.78	0.12	0.17	0.67
**Fe**	1.87	14	8.44	7.14	6.63	22.36
**Co**	1.54	1.45	1.99	1.25	0.70	0.07
**Ni**	1.65	2.01	2.24	1.19	0.95	0.38
**Cu**	2.57	2.46	2.21	2.47	2.72	1.03
**Zn**	0.63	0.58	0.50	<0.181	<0.181	<0.181
**As**	10.16	8.18	7.76	9.63	6.95	1.33
**Se**	5.05	5	3.11	1.54	0.75	<0.456
**Mo**	132.64	121.16	86.08	40.06	18.36	1.16
**Cd**	0.03	0.04	0.02	0.02	0.01	0.00
**Sb**	6.00	4.66	4.00	6.72	4.38	0.32
**Ba**	205.63	237.13	170.99	96.47	70.92	10.63
**Hg**	0.01	0.02	0.01	0.02	0.01	0.00
**Pb**	0.03	0.03	0.03	0.00	0.01	0.03

CFA: Coal-fly-ash.

Table S4. Chemical elements concentrations in leachates obtained from soil pots growing *B. juncea*.

B. juncea						
**Values in mg L^{-1}**						
**Soil media**	**WEEK 1**	**WEEK 2**	**WEEK 5**	**WEEK 7**	**WEEK 9**	**WEEK 11**
**Al**	1.79	2.64	0.89	0.67	0.52	0.32
**Ca**	29.50	30.88	5.73	110.40	3.32	0.99
**K**	28.49	25.39	11.45	48.95	7.37	0.72
**Mg**	16.29	17.70	2.33	60.30	2.44	0.33
**Na**	20.62	17.65	2.35	81.25	2.47	0.32
**P**	0.01	0.01	0.02	< 0.1	0.07	0.02
**Si**	11.58	12.52	1.90	159.80	32.58	0.94
**Sr**	0.17	0.19	0.04	0.76	0.03	0.01
**Values in μg L^{-1}**						
**Li**	<0.680	1.75	0.89	12.13	4.81	0.92
**Be**	0.05	0.04	0.06	1.96	0.42	<0.039
**B**	79.81	88.07	23.49	922.35	29.30	10.74
**Ti**	29.81	38.39	16.47	722.26	4314	12.85
**V**	2.70	4.03	1.83	61.38	27.87	1.33
**Cr**	1.65	2.52	0.94	41.20	20.02	0.66
**Mn**	8.38	5.11	85.83	72.55	33.10	4.35
**Fe**	779.13	1140.13	437.07	36620.00	11640.00	287.61
**Co**	0.89	1.12	0.62	6.02	2.61	0.08
**Ni**	7.06	6.26	2.32	45.65	18.72	0.85
**Cu**	7.66	6.64	3.18	28.16	10.47	1.37
**Zn**	3.54	3.43	1.49	24.86	11.01	0.88
**As**	0.64	0.67	0.54	2.45	1.09	0.28
**Se**	1.10	1.26	0.45	1.05	<0.456	<0.456
**Mo**	1.07	0.75	0.32	0.97	0.18	0.14
**Cd**	0.03	0.03	0.00	0.03	0.01	0.01
**Sb**	0.11	0.09	0.05	0.22	0.11	0.03
**Ba**	139.06	146.75	17.96	367.56	135.07	5.42
**Hg**	0.00	0.00	<0.004	<0.003	0.00	<0.003
**Pb**	0.18	0.24	0.07	6.02	1.73	0.05

Table S5. Chemical elements concentrations in leachates obtained from CFA pots growing *B. juncea*.

B. juncea						
**Values in mg L^{-1}**						
**CFA media**	**WEEK 1**	**WEEK 2**	**WEEK 5**	**WEEK 7**	**WEEK 9**	**WEEK 11**
**Al**	1.68	2.44	0.90	0.18	0.21	0.09
**Ca**	97.71	127.60	151.20	138.20	97.63	78.12
**K**	47.07	55.85	39.60	8.62	7.72	6.97
**Mg**	4.00	6.80	7.76	45.79	32.14	24.59
**Na**	31.67	40.40	19.22	9.22	8.10	7.20
**P**	< 0.005	0.01	< 0.005	0.03	0.04	0.03
**Si**	2.02	0.87	1.74	1.91	1.88	1.89
**Sr**	2.84	3.10	4.32	3.08	1.94	1.55
**Values in µg L^{-1}**						
**Li**	400.76	476.44	331.10	352.88	258.72	216.49
**Be**	0.00	0.01	0.00	<0.039	<0.039	<0.039
**B**	1365.75	1583.59	1491.62	6486.03	4901.60	4154.41
**Ti**	1.16	0.14	0.25	<0.070	<0.070	<0.070
**V**	54.04	71.46	66.39	25.92	25.28	25.03
**Cr**	62.75	64.82	74.56	29.15	18.61	13.04
**Mn**	0.92	0.44	0.89	1.07	0.75	0.48
**Fe**	35.56	3.12	1.56	1.13	0.93	0.62
**Co**	0.43	0.56	0.43	0.31	0.25	0.22
**Ni**	1.25	0.93	1.20	0.64	0.35	0.36
**Cu**	1.37	0.70	1.15	0.71	0.57	0.74
**Zn**	0.25	0.28	0.46	<0.181	<0.181	<0.181
**As**	3.42	8.56	5.42	6.06	5.86	5.17
**Se**	6.34	9.14	6.51	13.24	9.56	8.97
**Mo**	59.55	104.97	118.20	73.77	47.21	31.39
**Cd**	0.01	0.02	0.02	0.04	0.03	0.03
**Sb**	3.79	4.58	4.11	9.84	8.98	8.38
**Ba**	126.98	103.05	126.93	70.14	65.90	62.63
**Hg**	0.01	0.02	0.02	0.02	0.01	0.01
**Pb**	0.02	0.01	0.02	0.01	0.00	0.00

CFA: Coal-fly-ash.

Table S6. Chemical elements concentrations in leachates obtained from CFA + soil pots growing *B. juncea*.

B.juncea						
**Values in mg L^{-1}**						
**CFA + soil media**	**WEEK 1**	**WEEK 2**	**WEEK 5**	**WEEK 7**	**WEEK 9**	**WEEK 11**
**Al**	0.03	0.05	0.07	0.05	0.04	0.01
**Ca**	280.40	217.00	219.30	102.23	51.01	6.20
**K**	49.45	35.79	35.83	12.89	6.91	0.54
**Mg**	30.10	23.62	27.80	21.86	15.82	1.38
**Na**	49.82	29.15	19.87	15.22	6.13	0.44
**P**	0.03	0.06	0.04	0.04	0.04	0.04
**Si**	4.13	2.99	2.43	1.23	0.62	0.59
**Sr**	4.61	3.45	15	2.98	1.12	0.13
**Values in μg L^{-1}**						
**Li**	25.90	27.30	187.29	53.62	19.57	2.54
**Be**	0.01	0.00	0.01	0.04	<0.039	<0.039
**B**	1841.70	2140.25	2405.82	2100.15	1846.93	141.76
**Ti**	0.37	0.22	0.29	0.30	0.29	0.34
**V**	14.56	18.02	26.87	24.12	23.42	4.84
**Cr**	49.42	45.07	47.02	9.57	3.69	0.42
**Mn**	0.45	0.51	15.49	0.23	0.11	0.84
**Fe**	2.62	2.06	2.71	3.21	4.63	20.77
**Co**	1.78	1.51	2.90	1.23	0.74	0.06
**Ni**	2.78	1.29	2.64	2.03	0.93	0.32
**Cu**	4.67	1.60	4.11	3.20	2.78	0.98
**Zn**	1.42	0.30	0.81	0.62	<0.181	<0.181
**As**	11.04	10.36	7.83	8.21	7.20	1.33
**Se**	6.42	18	8.83	1.88	0.83	<0.456
**Mo**	189.41	132.89	163.92	68.25	18.74	1.07
**Cd**	0.03	0.02	0.03	0.02	0.01	0.01
**Sb**	5.24	6.15	6.89	6.32	4.46	0.32
**Ba**	235	180.79	208.23	126.24	71.63	10.94
**Hg**	0.02	0.02	0.03	0.01	0.01	0.00
**Pb**	0.04	0.02	0.04	0.02	0.01	0.03

CFA: Coal-fly-ash.

Table S7. Chemical elements concentrations in leachates obtained from CFA pots where no plants were grown.

No plants in the growth media						
**Values in mg L^{-1}**						
**CFA media**	**WEEK 1**	**WEEK 2**	**WEEK 5**	**WEEK 7**	**WEEK 9**	**WEEK 11**
**Al**	0.49	2.86	0.46	0.39	0.30	0.21
**Ca**	122.20	100.60	135.90	155.40	99.37	90.51
**K**	85.04	60.71	39.80	13.07	8.23	7.65
**Mg**	6.35	6.20	10.92	59.60	28.23	24.64
**Na**	78	49.17	21.15	11.11	6.36	5.76
**P**	0.01	0.01	0.00	0.03	0.01	0.03
**Si**	0.90	0.66	1.60	2.27	2.19	2.23
**Sr**	3.77	3.18	6	3.03	1.95	1.83
**Values in μg L^{-1}**						
**Li**	919.53	727.95	423.05	360.85	224.33	213.18
**Be**	0.00	0.00	0.00	<0.039	<0.039	<0.039
**B**	1544.72	1444.04	1826.02	6640.43	4556.76	4355.44
**Ti**	0.07	0.14	0.15	<0.070	<0.070	<0.070
**V**	75.56	82.59	75.19	30.13	25.87	26.84
**Cr**	111.31	98.10	112.31	24.13	15.19	12.91
**Mn**	0.96	0.67	1.24	7.32	39.83	0.17
**Fe**	0.59	1.15	0.73	0.92	0.55	0.52
**Co**	0.67	0.51	0.42	0.40	0.25	0.21
**Ni**	0.42	1.28	0.48	0.69	0.77	0.37
**Cu**	0.47	0.67	0.81	0.78	1.21	0.43
**Zn**	0.14	0.37	0.36	<0.181	<0.181	<0.181
**As**	8.47	9.39	6.42	10.79	6.63	6.05
**Se**	9.20	10.08	6.80	20.74	10.50	9.55
**Mo**	194.97	156	135.30	99.08	43.72	35.91
**Cd**	0.04	0.03	0.02	0.06	0.03	0.02
**Sb**	3.66	4.54	5.02	11.12	8.77	8.51
**Ba**	108.52	99.40	133.74	81.99	62.13	60.63
**Hg**	0.02	0.02	0.03	0.02	0.01	0.01
**Pb**	0.01	0.02	0.02	0.00	0.00	<0.002

CFA: Coal-fly-ash.

Table S8. BCF for chemical elements accumulating in *B. juncea* and *S. oleracea L* from 69 to 115 days for all the growth media.

B. juncea																		
	**69 days**									**115 days**								
	**Soil**			**CFA**			**CFA + Soil**			**Soil**			**CFA**			**CFA + Soil**		
	**Leaf**	**Root**	**Stem**	**Leaf**	**Root**	**Stem**	**Leaf**	**Root**	**Stem**	**Leaf**	**Root**	**Stem**	**Leaf**	**Root**	**Stem**	**Leaf**	**Root**	**Stem**
**B**	1.90	0.14	0.53	0.13	0.01	0.03	0.06	0.01	0.03	2.67	1.01	2.40	0.12	0.08	0.06	0.07	0.02	0.03
**Cr**	6.83	1.56	1.41	1.33	0.28	0.40	0.85	0.55	0.82	5.66	4.37	2.06	1.00	1.08	0.55	2.13	0.58	0.65
**Mn**	24.40	11.77	6.40	899.87	101.97	202.47	151.94	57.02	140.26	24.00	15.79	7.72	309.84	200.39	262.18	315.57	118.69	106.95
**Ni**	1.82	0.67	0.45	27.56	6.57	9.51	4.67	3.47	4.62	1.20	1.32	0.59	24.57	24.32	11.75	10.66	3.47	3.28
**Cu**	9.23	2.85	2.12	74.10	14.91	22.20	7.05	3.83	5.87	6.53	5.96	2.46	27.58	19.96	25.26	9.31	4.44	3.78
**Zn**	41.45	8.13	9.81	455.94	118.45	139.82	68.25	44.97	55.13	16.43	28.56	11.90	185.73	181.15	196.67	85.29	44.32	35.85
**Mo**	6.60	0.19	0.73	0.21	0.02	0.04	0.10	0.04	0.03	7.29	1.12	3.24	0.28	0.15	0.15	0.03	0.02	0.03
**Ba**	3.65	0.86	1.38	3.36	0.69	1.08	0.88	0.47	0.72	2.72	2.36	1.86	1.22	1.10	1.38	1.32	0.50	0.52
**Fe**	77.45	18.86	14.39	7774.27	578.01	1074.13	1873.53	684.67	2066.67	68.31	50.51	17.89	979.08	692.36	1098.15	4046.11	1128.22	1047.44

S. oleracea L																		
	**69 days**									**115 days**								
	**Soil**			**CFA**			**CFA + Soil**			**Soil**			**CFA**			**CFA + Soil**		
	**Leaf**	**Root**	**Stem**	**Leaf**	**Root**	**Stem**	**Leaf**	**Root**	**Stem**	**Leaf**	**Root**	**Stem**	**Leaf**	**Root**	**Stem**	**Leaf**	**Root**	**Stem**
**B**	0.16	0.10	0.11	0.01	0.01	0.01	0.01	0.01	0.01	0.11	0.11	0.11	0.01	0.01	0.01	0.01	0.01	0.01
**Cr**	1.08	1.13	1.15	0.37	0.38	0.38	1.24	1.24	1.24	1.13	1.13	1.13	0.38	0.38	0.38	1.24	1.24	1.24
**Mn**	9.62	6.55	6.22	105.31	95.16	96.08	8.13	7.95	8	6.92	6.89	6.90	97.36	97.28	97.32	8.01	8	8
**Ni**	0.52	0.53	0.50	11.29	11.29	11.18	6.11	6.10	6.09	0.51	0.51	0.51	11.24	11.23	11.23	6.10	6.10	6.10
**Cu**	2.33	1.99	1.77	18.47	17.55	17.37	9.45	9.33	9.34	1.94	1.93	1.93	17.62	17.61	17.61	9.36	9.36	9.36
**Zn**	7.66	5.33	5.21	46.04	42.01	42.53	93.17	91.38	91.86	5.67	5.65	5.66	42.96	42.93	42.95	91.92	91.91	91.92
**Mo**	0.30	0.22	0.24	0.01	0.01	0.01	0.01	0.01	0.01	0.24	0.24	0.24	0.01	0.01	0.01	0.01	0.01	0.01
**Ba**	0.45	0.32	0.31	0.51	0.47	0.48	0.36	0.35	0.35	0.34	0.34	0.34	0.48	0.48	0.48	0.35	0.35	0.35
**Fe**	0.23	0.25	0.19	990.45	979.55	931.66	259.83	257.75	255.96	0.22	0.22	0.22	957.95	957.33	957.31	257.23	257.19	257.20

BCF: Bioconcentration Factor; CFA: Coal-fly-ash.
